# Experimental Study of Flow Boiling Regimes Occurring in a Microfluidic T-Junction

**DOI:** 10.3390/mi14122235

**Published:** 2023-12-13

**Authors:** Xiangzhong Bao, Fei Yang, Xuan Zhang

**Affiliations:** 1 Southeast University Architectural Design and Research Institute Co., Ltd., Nanjing 210096, China; baoxiangzhong@outlook.com; 2 School of Energy and Environment, Southeast University, Nanjing 210096, China; leo.yang@nexteer.com

**Keywords:** microchannel, boiling, flow pattern, bubble

## Abstract

Microchannel flow boiling is an efficient cooling method for high-heat-flux electronic devices. To understand the flow boiling regime in a T-shaped microchannel, this paper prepared T-shaped microchannels of different sizes and designed an experimental platform for the visualization of flow boiling in microchannels, and aimed to study the evolution characteristics of two-phase flow patterns in T-shaped microchannels. The influences of the flow rate and channel size on the boiling flow pattern inside a T-shaped microchannel were experimentally observed and quantitatively described. The results indicate that the occurrence position of the vaporization core gradually migrates from branch channel to main channel as the wall temperature increases. The flow boiling at the bifurcation of the T-shaped microchannel mainly includes the extrusion fracture flow, bubble flow, plug–annular alternating flow and annular flow, in which the annular flow can be further divided into the intermittent annular flow and the stable annular flow. In addition, a high flow rate and small channel size can lead to the disappearance of the bubble flow, and the presence of the bubble flow delays the appearance of the annular flow.

## 1. Introduction

In recent years, with the development of science and technology, the development of high-tech, single-phase heat transfer has, in particular, become increasingly unable to meet the needs of production and life. For example, in the field of electronic heat dissipation, the heat dissipation per unit area changed from 1–100 W/cm^2^ and quickly soared to its current level of 1–100 W/cm^2^. The heat dissipation of electronic equipment used in some special fields even reaches 10^4^ W/cm^2^, which far exceeded the limit of the single-phase heat transfer. In order to solve these problems, the gas–liquid two-phase heat transfer with large latent heat handling, especially the flow boiling heat transfer, has become a hot topic for scholars to study. Although flow boiling is an effective method to solve the problem of heat dissipation with high heat flux, flow boiling is not a new research direction.

Flow boiling in microchannel (FBM) was introduced as a potential candidate for the heat dissipation of microelectronic and high-power components due to its superior thermal performance and compact structure [1,2,3]. The bifurcations are key elements in these microchannel devices for the two-phase fluid distribution and collection. In a microfluidic T-junction, the flow boiling involves bubble growth, deformation, breakup and coalescence, as well as instabilities in a confined micro structure, and experiences the complex process of two-phase flow, heat transmission and phase change, as well as interface evolution. During this process, the behavior of the boiling two-phase flow in a bifurcation has a close relationship with the channel dimension, mass and heat flux. In this context, comprehending the flow boiling mechanisms occurring in a microfluidic T-junction is quite important.

In microchannels, the surface tension force becomes the dominant force for the two-phase flow and FBM, while gravitational forces can be considered negligible, causing the flow boiling regime to be significantly different from the macroscale channels [4,5,6]. The scale effect on flow boiling has been examined experimentally and interpreted by numerical simulations. It is generally accepted that the typical patterns of the flow are slug flow and churn flow, as well as annular flow, which correspond to the region controlled by the surface tension, intermediate region and the region controlled by the inertial force for microscale flow boiling. We can confine the primary regimes of FBM to the nucleate boiling and convective evaporation as well as a combination of these two. In addition, the confined bubble (slug) flow [7,8], vapor back flow [9,10] and microbubble emission boiling [11,12] are observed in microchannels owing to the microscale effect. The above understanding of flow boiling dynamics and heat transfer regimes are related to the straight microchannel. The presence of bifurcation introduces more complex bubble interactions, flow instability and thermo-hydrodynamic coupling for two-phase FBM. However, up to now, how the bifurcation affects the two-phase FBM is less understood [13]. As a consequence, it is necessary to understand the flow boiling regimes in the T-junction microchannel, so as to reveal the flow performance as well as heat transmission regime during FBM with the T-junction.

Considering the potential application of bubble manipulation in micro engineering and technology, a great deal of efforts has been devoted to investigating the interfacial transport phenomena of the adiabatic two-phase flow in microchannels with a branching structure, especially the bubble breakup dynamics at the microfluidic T-shaped junction. Because of the bifurcation, the capillary pressure of the gas–liquid interface experiences a dramatic change during the splitting flow, which results in long-distance interactions among bubbles (slugs) downstream and upstream of the junction. As a result, the two-phase flow at the T-junction is complex and nonlinearly dynamic and possesses a chaotic feature for splitting flow behavior [14]. Studies have demonstrated that two-phase flow behaviors are dependent on a number of parameters, including the channel dimension, gas and liquid flow rate, confinement degree and capillary number. The typical flow regimes for the bubble passing through a T-junction are the breakup, obstructed, asymmetric and non-breakup types of tunnels [15,16]. The flow boiling regimes at a microfluidic T-junction are more complicated due to the multi-interaction among the dynamical evolution of the bubble (bubble formation, growth, collision, coalescence and breakup), the bifurcation effect and the interfacial heat and mass transport in a confined microspace. However, the detailed flow regimes for the boiling in the microchannel with a T-junction are unavailable in the literature. For purpose of understanding the FBM and heat transmission, a unified view of flow boiling regimes in a microfluidic T-junction is required.

Although a large number of studies exists on FBM [17,18,19,20,21], people have placed little attention on heat transfer regimes in microfluidic T-junctions. For the purpose of understanding the underlying features of two-phase FBM with the T-junction, we use a visualization experiment to study the heat transfer behaviors with the T-junction via the technology of high-speed photography. Based on this experiment, the influence of superheat degree and channel dimension on the flow boiling are examined and investigated. In addition, the heat transmission regimes observed in a microfluidic T-shaped junction are analyzed and discussed. Particularly, we present different flow patterns to quantitatively describe the flow boiling regimes, depending on the superheat degree and channel dimension.

## 2. Description of the Experiment

Aiming to elucidate the flow boiling regimes in microfluidic T-junctions, several microchannels configured with a T-shaped bifurcation are fabricated using glass, and then the flow pattern of boiling in these microchannels is experimentally investigated by a visualization system.

### 2.1. Experimental Setup and Procedure

**Figure 1:**
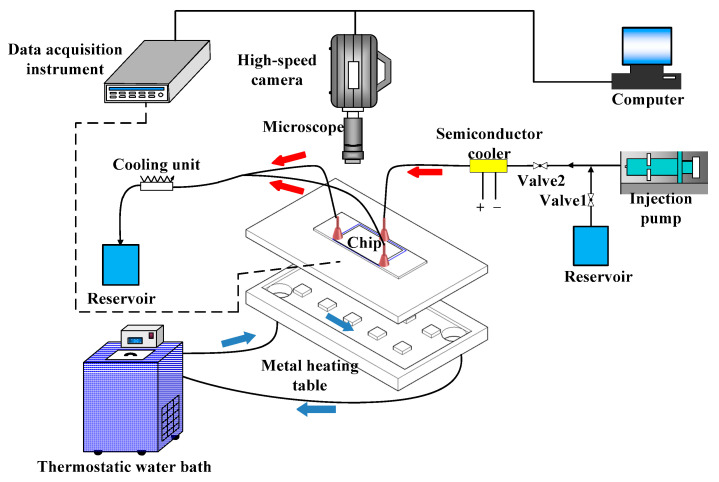
Experimental diagram of flow and boiling in T-shape microchannels.

**Figure 2:**
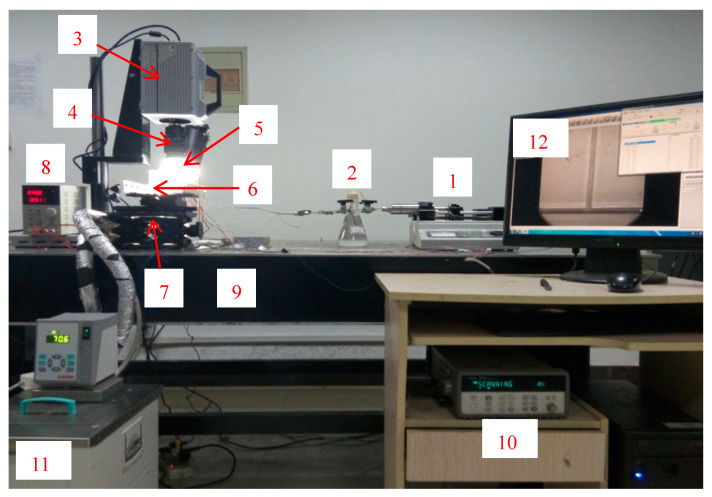
Diagram of flow and boiling in T-shape microchannels (1. Injection pump; 2. Reservoir; 3. High-speed camera; 4. Microscope; 5. High frequency no flashing light; 6. Experimental section; 7. Three-dimensional mobile platform; 8. DC power supply; 9. Optical experimental platform; 10. Agilent data acquisition device; 11. Thermostatic waterbath; 12. Computer).

The visualization experiment platform of flow boiling in T-shaped microchannels mainly consists of five parts: a liquid flow control and output device, an experimental section, a heating device, a high-speed microscopic imaging system and a data acquisition system. Figure 1 depicts the system diagram of the flow and boiling experiment conducted in T-shaped microchannels. Additionally, Figure 2 illustrates the physical diagram of the system. It can be observed that an injection pump is used to pump the working medium into the experimental section, and the working medium reaches the saturation temperature under the heating device and produces the boiling phenomenon. Meanwhile, the pictures of the FBM are collected by the high-speed microscopic imaging system; then, we save them in the computer, and the data acquisition system collects the temperature signals of each temperature measuring point arranged and saves them. Each component of the experimental system is described below.

#### 2.1.1. Liquid Flow Control and Output Device

**Figure 3:**
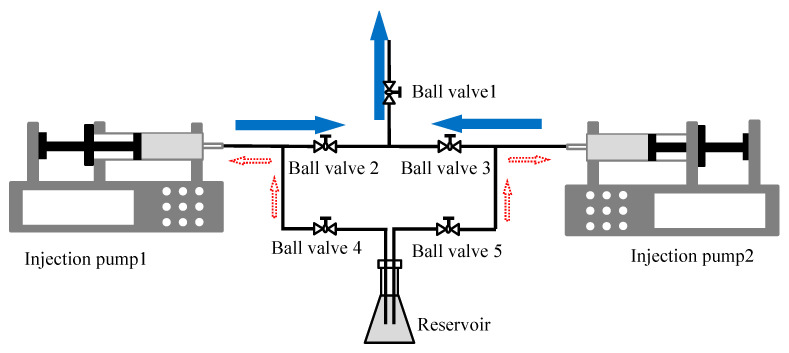
Schematic diagram of liquid flow control and output.

To achieve a continuous and constant flow of working fluid (refrigerant R141b) for the entire experimental system, two injection pumps, several stainless-steel pipelines, and several one-way ball valves are used in the experiment to form the liquid flow control and output device of the entire system, as can be observed in Figure 3. With the connection method shown in the figure, this device can provide uninterrupted and constant liquid supply during the experiment.

**Figure 4:**
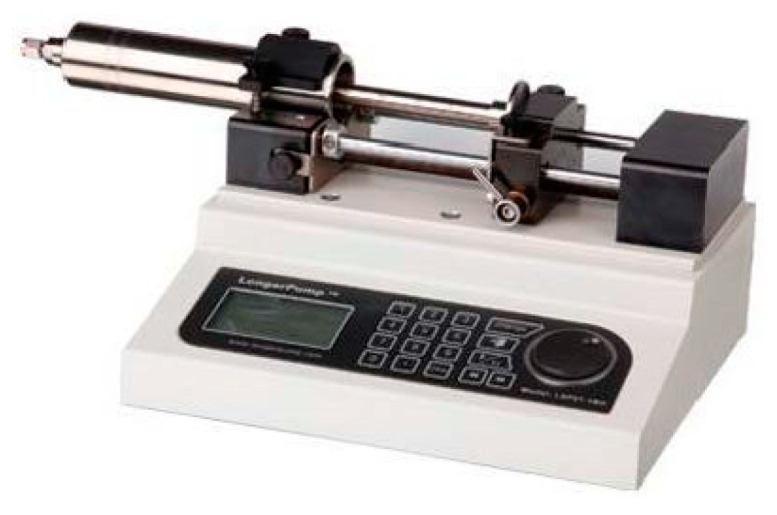
LSP01-1BH injection pump.

**Figure 5:**
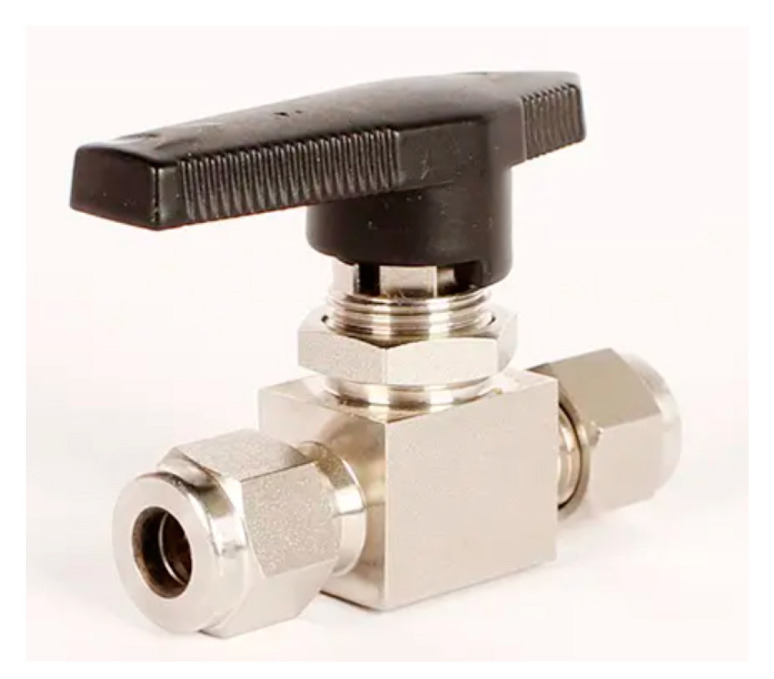
A 304 stainless steel ball valve (3 mm).

The injection pump used in the experiment is the laboratory-type injection pump LSP01-1BH produced by Baoding Lange Constant Flow Pump Co., Ltd. (Baoding, China), as shown in Figure 4. This injection pump has five working modes: injection, extraction, injection before extraction, extraction before injection, and continuous mode. The injection pump uses a built-in stainless-steel syringe with a maximum capacity of 50 mL and can control the flow error within a range of ±0.5%. The ball valve used in the experiment is a 304 stainless steel clamp ball valve, as shown in Figure 5. The pipe connected to this ball valve is a 304 stainless steel pipe (outer diameter: 3 mm; inner diameter: 2 mm).

In the experiment, the continuous and constant flow supply of the entire experimental system is achieved through the following steps:

(1) Before the experiment, close ball valves 1, 2 and 3. Open ball valves 4 and 5, and use injection pump 1 and injection pump 2 to extract the working fluid from the reservoir to full capacity, as shown by the red dashed arrow in Figure 2 and Figure 3.
(2) Before starting the experiment, close ball valves 3, 4 and 5. Open ball valves 1 and 2, and turn on the switch of injection pump 1. Injection pump 1 starts to inject the working fluid into the experimental section. When the injection volume reaches 95% of the full capacity, open ball valve 3 and the switch of injection pump 2, and simultaneously close ball valve 2 and injection pump 1. Injection pump 2 then injects the working fluid into the experimental section, while injection pump 1 stops working.
(3) Open ball valve 4, use injection pump 1 to extract the working fluid from the reservoir to full capacity, and then close ball valve 4. When the injection volume of injection pump 2 reaches 95% of the full capacity, open ball valve 2 and the switch of injection pump 1, and simultaneously close ball valve 3 and injection pump 2. At this time, injection pump 1 injects the working fluid into the experimental section, while injection pump 2 stops working.
(4) Open ball valve 5, use injection pump 2 to extract the working fluid from the reservoir to full capacity, and then close ball valve 5. When the injection volume of the injection pump 1 reaches 95% of the full capacity, repeat the process of steps 2, 3 and 4.

#### 2.1.2. Experimental Section

The experimental section mainly consists of an inlet liquid temperature control device, a T-shaped microchannel chip, and several inlet and outlet pipelines. The inlet liquid temperature control device is mainly composed of a semiconductor refrigeration chip and a DC power supply. Since we use R141b as the refrigerant herein, the saturation temperature at a standard atmospheric pressure is 32.05 °C. In order to maintain the same subcooling of the inlet liquid under all operating conditions, the temperature of the inlet liquid is controlled at 20 °C using the semiconductor refrigeration chip.

**Figure 6:**
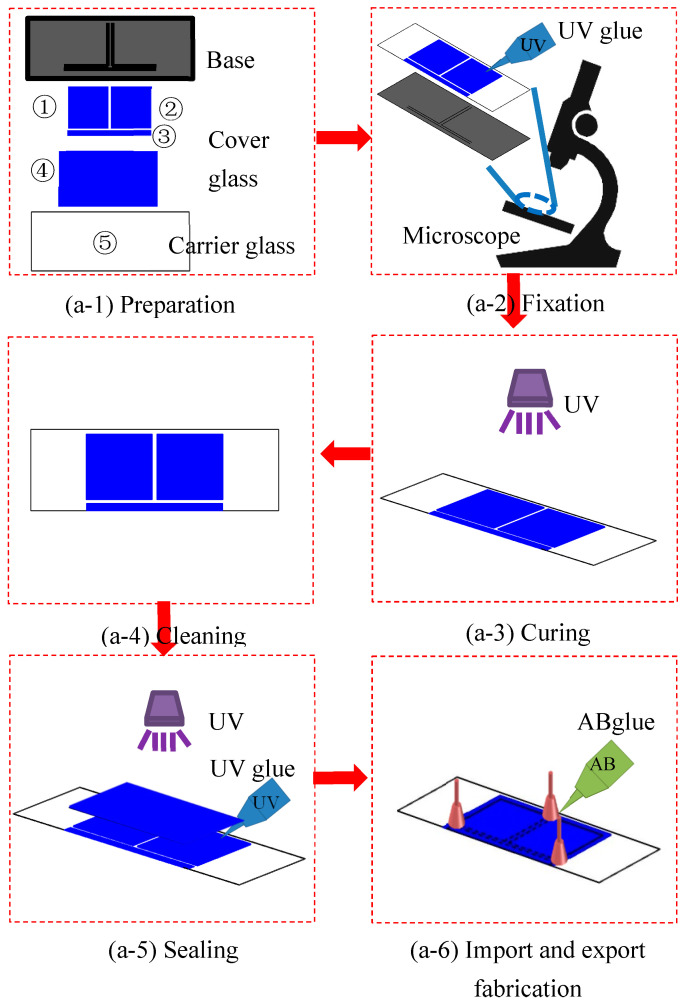
Production process of T-shaped microchannel chip.

The typical technologies for fabricating a microchannel, including silicon wafer etching [22,23], glass etching [24,25] and soft lithography in PDMS [26,27], are able to control the channel size precisely; however, the production processes are very complicated and costly because they fabricate well only with complex manipulations using an expensive experimental apparatus in a clean room. To achieve the convenient laboratory supply, Deng et al. [28] report a straightforward and cost-effective method for fabricating microfluidic devices to produce uniform emulsions using patterned coverslips and microscope glass slides. In this study, this approach is applied to fabricate the microchannel configured with a T-shaped bifurcation (referred as a T-shaped microchannel in the following) for the boiling experiment. As shown in Figure 4, the fabrication process can be listed as follows:

(1) Preparation stage: First, use drafting software such as AutoCAD 2017 to draw the shape of the microchannel. Then, print it as a substrate using a high-resolution printer, as shown in Figure 6(a-1). Cut the prepared cover glass (①, ②, ③ and ④) and carrier glass (⑤) into the desired dimensions using a glass cutter. Clean the cut glass slides by immersing them in alcohol. This is followed by rinsing with deionized water, and, finally, air drying.
(2) Fixation stage: Place the prepared substrate on the microscope stage and secure it in position. Place the clean carrier glass (⑤) on top of the substrate, aligning it under the microscope, and fix it in position. Then, using the microscope, align and fix the cover glasses (①, ②, ③) along the lines of the substrate to form the shape of the T-shaped microchannel.
(3) Curing stage: With the alignment completed, fill the contact surfaces with a UV glue (Guangzhou Hengda New Materials Technology Co., Ltd. (Guangzhou, China), K3307) using capillary action. Then, expose the contact surfaces to UV light for approximately 2 min to ensure a tight cure.
(4) Cleaning stage: Rinse the cured semi-finished chip with deionized water twice and allow it to air dry.
(5) Sealing stage: Seal the cleaned semi-finished chip with a clean cover glass (④). Fill the contact surfaces with UV glue using capillary action and expose it to UV light for approximately 2 min.
(6) Inlet and outlet fabrication: Finally, place a needle (outer diameter: 9 mm; inner diameter: 0.6 mm) at the entrance and exit of the T-shaped microchannel as the interface for connecting the microchannel and polyethylene tubing. Depending on the requirements, a thermocouple can be placed to monitor the inlet and outlet temperature. Use a modified acrylic acid ester adhesive (GLH-302) to secure the needle to the T-shaped microchannel chip.

In order to conduct comparative experiments, we designed T-shaped microchannels of different sizes to study the flow pattern evolution and temperature instability under different operating conditions. The T-shaped microchannel consists of a main channel and left and right branch channels. The length of which are *L*_1_ and *L*_2_, respectively, and the width of which are *w*_1_ and *w*_2,_ respectively. The depth of all channels is *h*. Four types of T-shaped microchannels were designed for visual experiments on flow boiling. Among them, the first, second, and third types of T-shaped microchannels have the same channel dimensions, and only the depth dimension *h* is varied. The fourth type of T-shaped microchannel is designed to be closer to conventional channels, with specific dimensions shown in Table 1 and Table 2.

#### 2.1.3. Heating Unit

During the experiment, a thermostatic water bath was mainly used to heat the T-shaped microchannel chip. The main method is as follows: the inlet and outlet of the constant temperature water bath are connected to a metal heating block, and the constant temperature water bath provides a continuous supply of constant temperature hot water to heat the metal heating block. Through the combined effects of convection and conduction heat transmission, the upper surface of the metal heating block maintained a constant temperature, thereby providing a constant temperature heating condition to the T-shaped microchannel chip.

**Figure 7:**
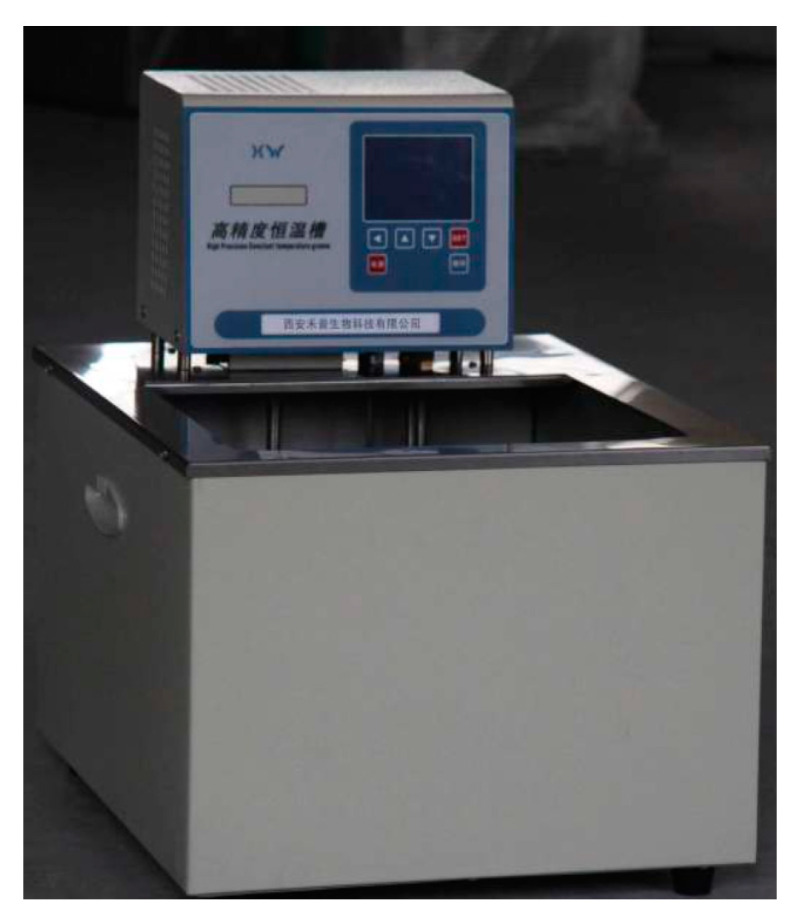
Physical diagram of MC JULABO immersion heating circulator.

The constant temperature water bath used in the experiment is the JULABO immersion heating circulator (MC type), as shown in Figure 7. The MC-type JULABO immersion heating circulator adopts an ultra-quiet design and can operate in an ambient temperature range from 5 to 200 °C. The temperature stability of the provided constant temperature water is about ±0.01 °C, and the heating power is 2 kW. At the same time, the pressure pump pressure can be adjusted, providing variable flow rate (11–16 L/min) functionality.

**Figure 8:**
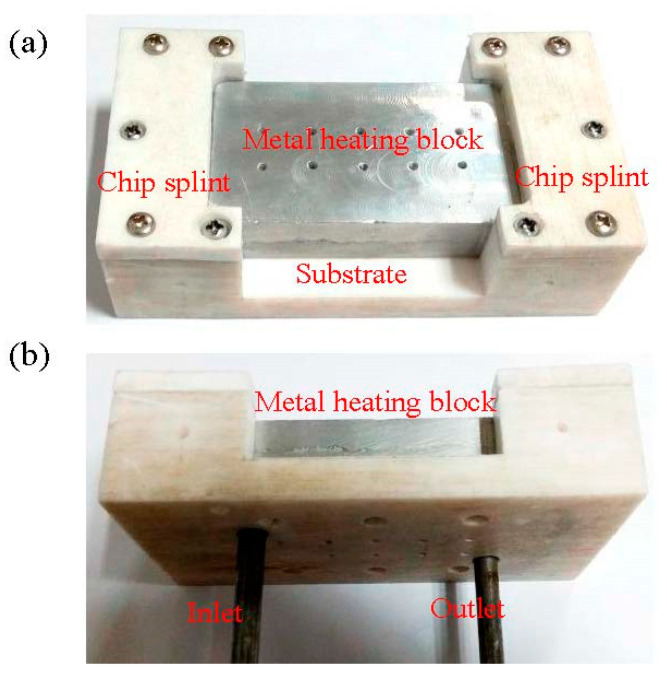
Physical diagram of metal heating block. (**a**) front; (**b**) side.

**Figure 9:**
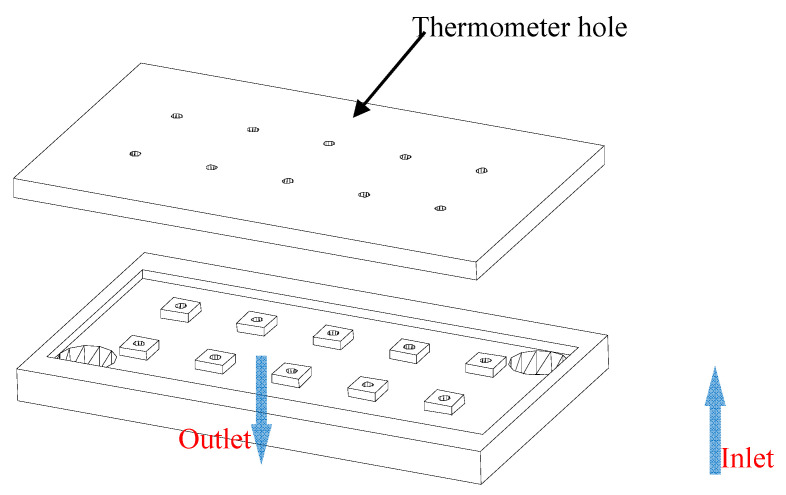
Three-dimensional section view of metal heating block.

The metal heating block is an aluminum block with internal channels for processing, and it is connected to two pipes as the inlet and outlet joints. The physical appearance of the metal heating block is shown in Figure 8, and the 3D sectional view is shown in Figure 9. There are also 10 temperature measurement points arranged on the metal heating block to monitor its temperature and uniformity. The metal heating block is fixed on a PTFE base, and two PTFE chip clamps are used to secure the T-shaped microchannel chip. During the experiment, thermal conductive grease should be applied on the contact surface between the T-shaped microchannel chip and heat source to ensure that the temperature is consistent with that of the T-shaped microchannel chip.

#### 2.1.4. High-Speed Microscopic Imaging System

**Figure 10:**
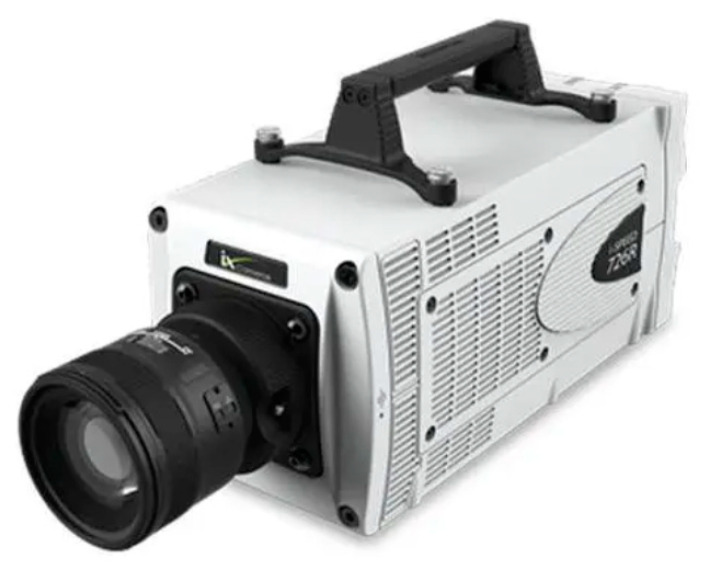
High-speed camera: Photron FASTCAM SA4.

**Figure 11:**
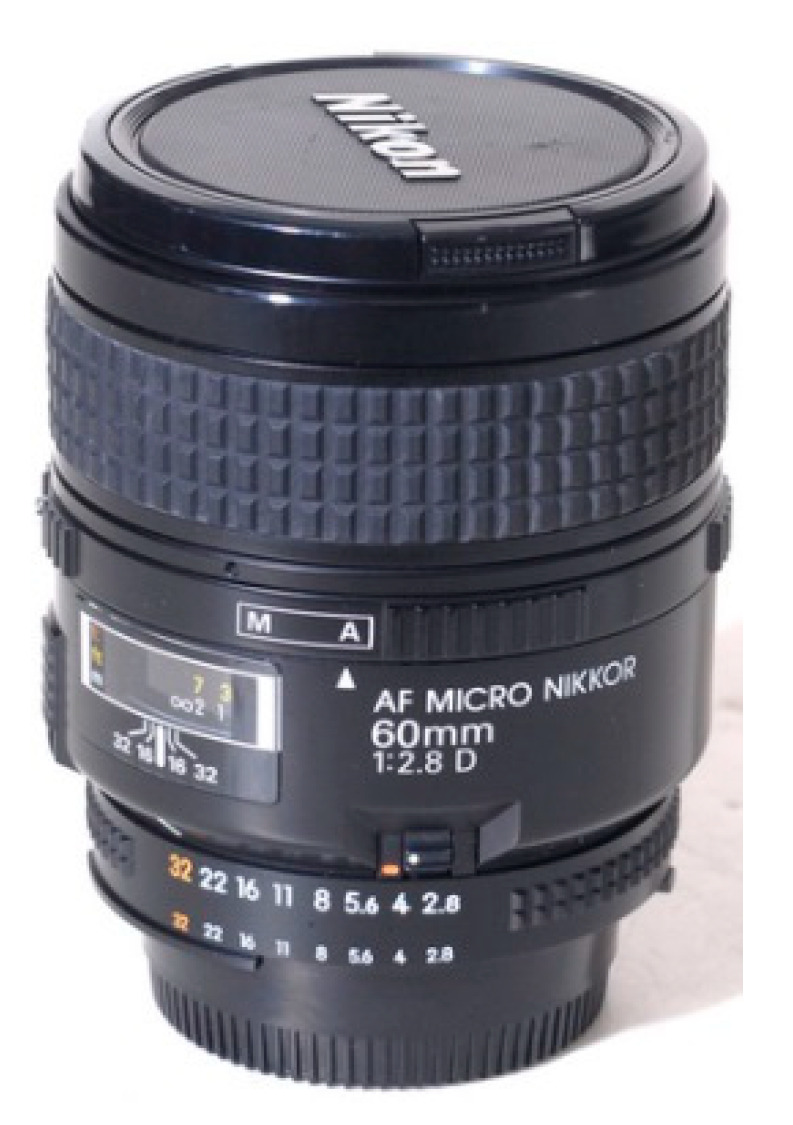
Nikon microlens.

In order to observe the flow boiling phenomenon clearly, a combination of a high-speed camera CCD (presented in Figure 10), microscope lens (as shown in Figure 11), and auxiliary light source is used to capture and store real-time images of the flow boiling phenomenon. The CCD utilized has a resolution of 1024 × 1024 pixels, covering the full frame, and a maximum shooting speed of 3600 fps. The maximum shooting speed in a segmented mode can reach 500,000 fps, and the fastest shutter speed can reach 1 μs. In the experiment, the shooting speed is generally set to 1000 fps~3000 fps. In order to capture the flow boiling phenomenon in the microchannel more clearly, a microscope lens is used for magnification. The maximum magnification of this microscope lens is 32 times. Due to the small aperture of this model of high-speed camera CCD, the lighting effect is poor. Therefore, an auxiliary light source, namely a high-frequency non-flashing lamp, is needed to increase the brightness of the object being captured. The best shooting effect can be achieved by adjusting the incident angle of the auxiliary light source.

#### 2.1.5. Data Acquisition System

**Figure 12:**
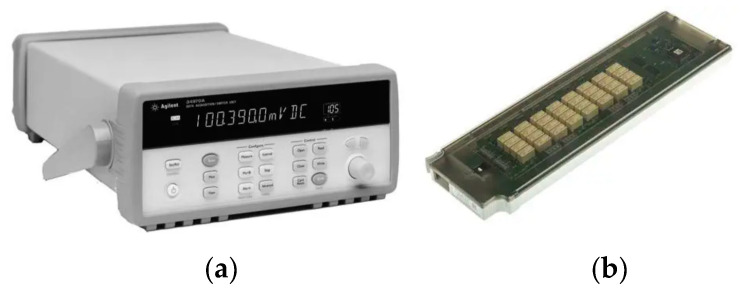
Physical diagram of data acquisition instrument. (**a**) Data acquisition unit; (**b**) data acquisition board.

The system mainly includes a thermocouple, data acquisition instrument and computer. The thermocouple used is a copper-constantan thermocouple produced by OMEGA, a company in the United States. The thermocouple is a T-type and has a wire diameter of 0.1 mm. The maximum long-term measurement temperature is 150 °C, and the measurement accuracy is ±0.1 °C. The data acquisition instrument used is Agilent 34970a, which is produced by Agilent Technologies, a company in the United States, as can be seen in Figure 12. This model of a data acquisition instrument consists of a data collector and data acquisition board. During the experiment, the positive and negative terminals of the T-type thermocouple are fixed to the data acquisition board through slots. The data acquisition board is inserted into the data acquisition instrument, and the data acquisition instrument converts the potential signal of the T-type thermocouple into a temperature signal., which facilitates the monitoring and recording of the temperatures at various measurement points during the experimental process.

#### 2.1.6. Experimental Method and Procedure

To study the flow and heat transmission phenomena of boiling flow in T-shaped microchannels, we use a high-speed microscopy imaging system to assist in observing the patterns and its evolution. At the same time, a data acquisition system is used to monitor and record the temperature of the entire experimental system. During the experiment, flow boiling experiments are conducted on four different sizes of T-shaped microchannels. The specific experimental steps are as follows:

(1) Develop an experimental plan, clarify the physical quantities to be measured and the flow boiling flow pattern images to be taken during the experiment, determine the various operating conditions that need to be tested, and create tables or other records as required.
(2) Produce the four T-shaped microchannels used in the experiment according to the process presented in Figure 6.
(3) Connect the various components according to the experimental system diagram and set up the high-speed camera microscopy system and the three-dimensional mobile platform on the optical experimental platform. Connect the inlet and outlet of the thermostatic water bath and the entrance and exit of the metal heating table with plastic hoses, and insulate the pipelines. Finally, check the air tightness of the entire experimental system.
(4) Turn on the power of the syringe pump, high-speed camera, constant temperature water bath, data acquisition device and computer, and check whether each component is working properly. Extract the working fluid with both syringe pumps to their full range. Before the experiment starts, check whether all thermocouple readings are normal. Turn on the high-speed camera, and adjust the magnification, shooting frequency, field of view size, and other parameters of the microscope. Then, start the syringe pump to purge the air in the entire experimental system and prepare for the experiment.
(5) Adjust the flow rate of the injection pump for the operating conditions, and change the temperature of the constant temperature water bath to conduct the experiment, monitoring the temperature displayed on the computer. After the temperature of the metal heating table stabilizes, capture the images of the flow pattern using the high-speed microscopy system. Temperature data are collected by the data acquisition system as needed.
(6) After the completion of a set of experiments with a fixed flow rate and different temperatures, wait for the T-shaped microchannel chip to cool to room temperature before adjusting the flow rate of the injection pump for the next set of experiments. Repeat steps (5) and (6).
(7) After the experiment is finished, turn off the CCD, the power of the constant temperature water bath, and the data acquisition device. Close the syringe pump after the T-shaped microchannel chip has cooled down.
(8) Clean up the experimental platform, and disassemble and clean the T-shaped microchannel chip. Replace the chip with a different size and repeat steps (3) to (8).
(9) Organize and analyze the experimental data and the obtained flow pattern images.

#### 2.1.7. Error Analysis

The main parameters involved in the experiment are the inlet mass flow density, temperature of the left and right branches of the T-shaped microchannel, and the heating temperature of the chip. The errors of these parameters are as follows:

(1) Temperature measurement error: In the experiment, temperature is measured using a T-type thermocouple with a single wire diameter of 1 mm. After correction using a constant temperature water bath ice point, the measurement error δ*T* of the temperature measuring instrument is ±0.1 °C. The lowest measured temperature value T in this experimental section is 20 °C, and the maximum relative error of the temperature measured by the instrument is δ*T*/*T*_min_ = 0.5%.
(2) Mass flow density measurement error: Based on the accuracy of the syringe pump used, the error range of the mass flow density *G* is ±0.5%.
(3) Image quantization error: In this experiment, the resolution of all captured images is 72 ppi, which means there are 72 pixels per inch (1 inch = 25.4 mm). The error in selecting the number of pixels by the pixel software is 1, so the relative error of the position data quantized from the captured images is 38%.

## 3. Results and Discussions

According to the existing research on FBM, the main focus is on the instability of the flow boiling phenomena and temperature-related parameters on a microscale. In this chapter, experimental research on the flow boiling of refrigerant R141b in T-shaped microchannels is conducted. Experiments are carried out in four different sizes of T-shaped microchannels. The main observed phenomena during the experiments include bubble nucleation, growth, coalescence, sliding, and detachment from the wall. These basic bubble behaviors can evolve into various flow boiling flow patterns. The main flow boiling patterns in conventional channels are bubble flow and slug flow, as well as annular flow. Because of various factors affecting flow boiling, such as wall roughness, fluid velocity, channel shape, and size, flow boiling phenomena exhibit strong randomness and complexity. Moreover, compared to conventional channels, the flow range in microchannels is more narrow, making the variation of flow boiling flow patterns more complex. The features of flow boiling and heat transmission in T-shaped microchannels will be discussed from three aspects: bubble dynamics, flow boiling flow patterns and their evolution at the bifurcation, and the instability of outlet temperature.

### 3.1. Bubble Behavior in T-Shaped Microchannel Flow Boiling

#### 3.1.1. Bubble Generation

**Figure 13:**
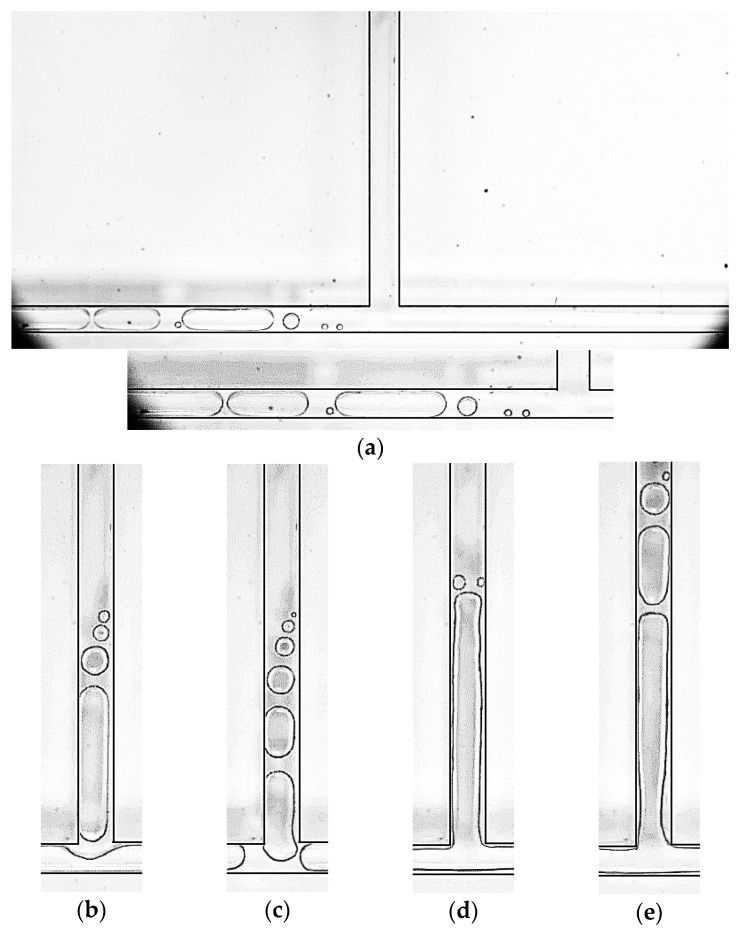
Nucleated bubbles appear at different locations in the channel, in working conditions: Type 2, *G* = 113.6 kg/m^2^·s, (**a**) *T*_heat_ *=* 37.5 °C, (**b**) *T*_heat_ *=* 40°C, (**c**) *T*_heat_ *=* 42 °C, (**d**) *T*_heat_ *=* 46 °C and (**e**) *T*_heat_ *=* 54 °C.

During the experiment, the subcooled fluid (liquid) enters the chip through the pipeline, and the chip is in close contact with a temperature-controlled metal heating platform, with thermal grease filling the contact surface. When the flow in the entire experimental system reaches a steady state, it can be considered as a continuous flow of subcooled fluid passing through the temperature-controlled heating surface, being continuously heated, and the temperature of the microchannel and the fluid will continuously increase. When the temperature reaches a certain level, nucleation bubbles will appear, indicating the occurrence of nucleate boiling. Since the fluid is continuously heated, there is a certain temperature gradient along the flow direction. Therefore, nucleation bubbles will first appear in the branch channel during the experiment, and then in the main channel. We can find in Figure 13 that, in the type 2 chip, under the condition that the mass flow rate of the main channel is 113.6 kg/m^2^·s, nucleation bubbles first appear in the branch channel. As the channel wall temperature increases (Figure 13b–e), the location of nucleation bubbles moves from the branch channel to the main channel, getting closer to the inlet of it. This phenomenon of nucleation core movement is particularly evident under a high mass flow rate density. Under low mass flow rate density, the temperature gradient decreases due to the slower flow velocity of fluid, making it easier to reach thermal stability. Therefore, the initial position of nucleation cores appears in the main channel.

#### 3.1.2. Combination of Bubbles

**Figure 14:**
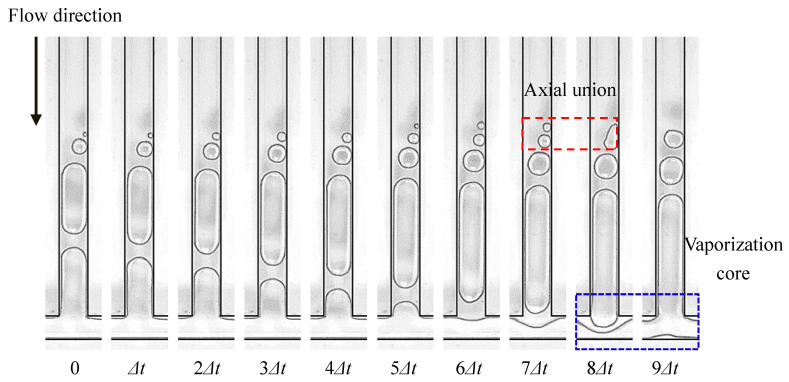
Axial merging of bubbles, Δ*t* = 2 ms, and working condition: Type 2, *G* = 113.6 kg/m^2^·s and *T*_heat_ *=* 37.5 °C.

**Figure 15:**
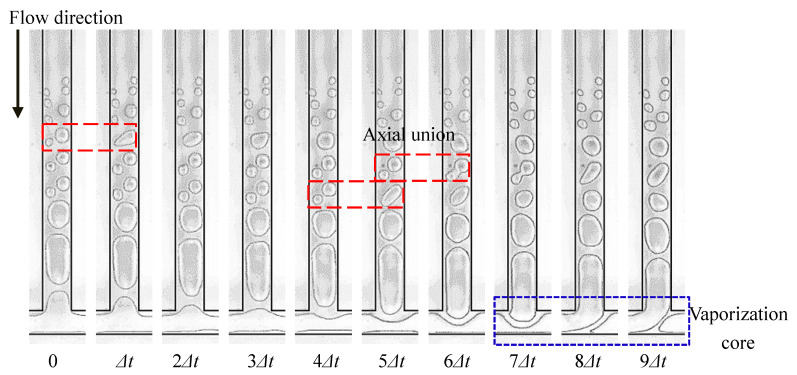
Normal merging of bubbles, Δ*t* = 0.33 ms, and working condition: Type 2, *G* = 113.6 kg/m^2^·s and *T*_heat_ *=* 45.8 °C.

Bubble coalescence is the most typical behavior of bubbles during the FBM. After the appearance of nucleation bubbles in the boiling nucleus, the vapor–liquid interface of the bubbles absorbs heat and evaporates, increasing the gas volume inside the bubbles, which is the process of bubble growth. Nucleation bubbles grow in the direction of the flow, and in the normal direction, as they flow with the mainstream. If there are two or more adjacent nucleation nuclei on the same side wall, the nucleation bubbles generated by these nuclei will grow in the axial direction. When the volume of the bubbles reaches a certain level, these two nucleation bubbles come into contact and collide, merging into a larger bubble under the influence of surface tension. This phenomenon is called an axial coalescence of bubbles, as shown in Figure 14. If there are nucleation nuclei on both side walls, the nucleation bubbles they generate will also collide and merge in the normal direction. This merging phenomenon is called the normal coalescence of bubbles, as shown in Figure 15. Before the nucleation bubbles detach from the boiling nucleus, the bubble interface absorbs heat from the wall, which also proves that bubbles enhance the heat transmission in nucleate boiling, and the coalescence of bubbles helps the nucleation bubbles detach from the boiling nucleus, thus generating new nucleation bubbles faster and further enhancing the heat transfer.

In addition, the coalescence between bubbles and plugs, between plugs and between plugs and annular flows were observed. These coalescence phenomena are the basis for the evolution of flow patterns of FBM. For example, the coalescence of bubbles will cause a further growth of the bubbles. The vapor–liquid interface formed after the coalescence continues to absorb heat and evaporate, increasing the gas volume. When the normal direction of bubble growth is restricted by the width of microchannels, the bubbles can only grow along the axial direction, which change from initially spherical to cylindrical with spherical ends; this is one of the formation modes of plugs. Similarly, the coalescence of multiple plugs in microchannels is also one of the ways in which the slug flow transitions to annular flow.

#### 3.1.3. The Rupture of the Bubble

The rupture of bubbles in T-shaped microchannels is a unique bubble dynamics phenomenon caused by the presence of bifurcation structures. Generally speaking, during FBM or parallel microchannels, bubble coalescence is the main phenomenon observed, and bubble rupture is not observed. However, in T-shaped microchannels, when bubbles or plugs in the main channel pass through the bifurcation, they exert a squeezing effect on the plugs in the branch channel. The plugs in the branch channel may either merge with the bubbles or plugs in the main channel or rupture under the squeezing effect. As shown in Figure 14 from *t* = 8Δ*t* to *t* = 9Δ*t*, a T-shaped plug is formed by the merging of a plug in the main channel and a plug in the branch channel at the bifurcation. In Figure 15, during the time period from *t* = 7Δ*t* to *t* = 9Δ*t*, the plug in the branch channel is squeezed and ruptures into two sub-plugs. The sub-plug in the left branch then merges with the plug in the main channel to form an L-shaped plug. This situation of rupture and merging forms the characteristic flow pattern of FBM. In studies of two-phase flows without a phase change, it has been found that the merging and rupture of bubbles at the bifurcation are mainly related to the critical pressure and the length of the plug. However, in flow boiling processes, the merging and rupture of bubbles at the bifurcation are more complex than without the phase change, because the plug length and the pressure inside the channel change in real time.

### 3.2. The Flow Boiling Flow Pattern and Its Evolution at the Bifurcation of T-Shaped Microchannel

From existing studies, it can be found that in straight microchannels, the subcooled liquid enters the channel and is continuously heated during the flow process. When the liquid temperature approaches the saturation temperature, axial temperature gradients contribute to the formation of nucleation bubbles on the channel wall. This is known as subcooled boiling. As the liquid continues to flow, the bulk temperature reaches the saturation temperature, resulting in saturated boiling. During these two stages, only small bubbles are generated on the channel wall, so the flow boiling flow pattern is mainly in the form of a bubble flow. These small bubbles continue to flow and are heated, resulting in an increase in the bubble volume and occupation of the central part of the channel. As the slug flows continue to flow and are heated, they merge with each other to form a slug flow pattern. Finally, an annular flow pattern is formed, where only a ring-shaped liquid film flows around the channel wall. Because of the ongoing evaporation of the liquid film, it becomes thinner until it completely disappears, resulting in a dryout phenomenon. Therefore, flow patterns of FBM mainly include bubble flow, slug flow, annular flow, dryout flow, and so on. The main focus is on the effect of the bifurcation structure on the flow patterns of FBM with T-junction; thus, we mainly focus on the flow patterns at the bifurcation. Through visual experimental research, it can be found that the flow patterns at the T-junction of the microchannel mainly include the bubble flow, squeeze and rupture flow, slug–annular alternating flow and annular flow. During the experiment, a dryout flow was also observed. However, due to the rapid increase in wall temperature after the dryout, considering the heat resistance of the experimental glass chip and the safety of the entire experiment, the dryout flow was not allowed to occur for a long time during the experiment, and the dryout phenomenon is not discussed in this paper. Below, we will specifically discuss the flow boiling flow patterns at the bifurcation.

#### 3.2.1. Squeeze and Rupture Flow

The squeeze and rupture flow is a special flow pattern that occurs during FBM with the T-junction. Because of the presence of bifurcation, bubbles (plugs) will inevitably merge or rupture when passing through the bifurcation, forming the unique squeeze and rupture flow pattern in T-shaped microchannels.

**Figure 16:**
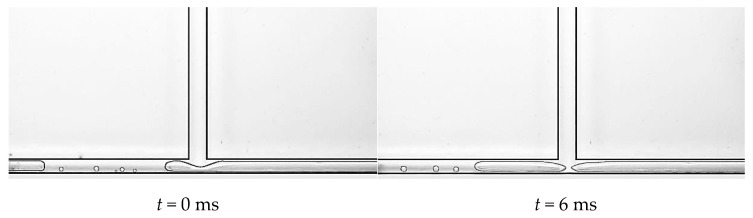
Squeeze and rupture flow, working condition: Type 2, *G* = 14.2 kg/m^2^·s and *T*_heat_ = 39.6 °C.

During the experiment, we mainly observed two situations of the squeeze and rupture flow. One situation is that at low mass flow density, as can be seen in Figure 16, the primary channel is single-phase liquid flow, while nucleation bubbles appear in the branch channel. The nucleation bubbles in the branch channel quickly grow into plugs, which occupy the branch channel. Due to the fluctuation of the outlet pressures, the plug pulsates back and forth in the branch channel, causing a blockage to the liquid flow in the primary channel at the junction. The accumulation of liquid in the main channel exerts squeezing pressure on the front end of the plug, causing the plug to neck down at the bifurcation, and eventually the plug ruptures into two subplugs. This is the most common form of the squeeze and rupture flow at the bifurcation of T-shaped microchannels, and it lasts for a relatively long time.

**Figure 17:**
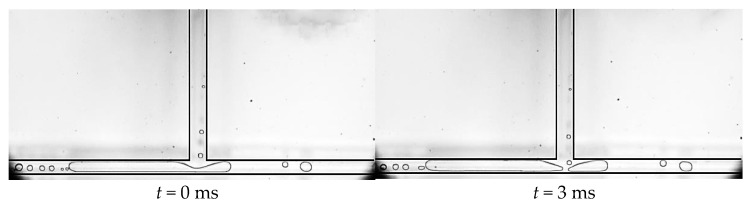
Squeeze and rupture flow, working condition: Type 2, *G* = 92.3 kg/m^2^·s and *T*_heat_ = 39.8 °C.

The other situation is that there are only a few circular nucleation bubbles that gradually grow with the main flow in the main channel, and plugs and circular small bubbles are distributed alternately in the branch channel, as shown in Figure 17. The bifurcation is occupied by alternating plugs in the branch channel and the liquid containing a small number of circular bubbles. When the plug blocks the bifurcation, the front end of the plug is pressurized and ruptures into a circular or slug bubble due to the pressure from the liquid containing circular bubbles in the main channel. At the bifurcation, there is also merging between the circular small bubbles in the main flow and the slug or circular bubbles in the branch channel. When the liquid containing circular bubbles is present at the bifurcation, the circular bubbles in the main channel directly enter the branch channel with the main flow. This situation mainly occurs when the mass flow density is relatively high and the nucleation bubbles in the main channel enter the bifurcation in the form of small bubbles. If the mass flow density is too low at this time, the nucleation bubbles in the main channel will enter the bifurcation via plugs, forming a slug flow pattern. This flow pattern can also be treated as a common flow pattern from the squeeze and rupture flow to the bubble flow.

#### 3.2.2. Slug Flow

**Figure 18:**
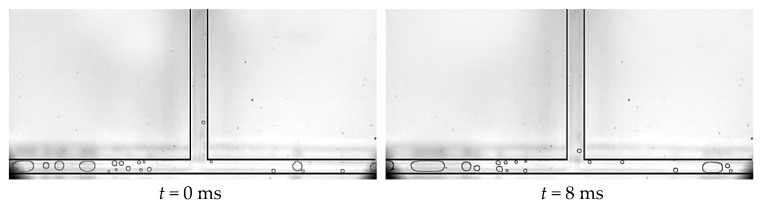
Slug flow, working condition: Type 2, *G* = 113.6 kg/m^2^·s and *T*_heat_ = 46.7 °C.

Slug flow is a typical flow pattern of FBM, and the main characteristics of slug flow at the bifurcation in T-shaped microchannel flow boiling are that there are only a few circular nucleation bubbles that grow gradually with the main flow in the main channel, while there are dispersed bubbles flowing downstream in the left and right branch channels, as shown in Figure 18. A few nucleation bubbles form in the main channel and grow gradually with the main flow, directly entering the branch channels at the bifurcation without a bubble rupture or coalescence. Most nucleation bubbles are generated in the branch channels, and their growth rate is higher than that in the main channel. They grow into slug bubbles in the downstream of the branch channels, changing from circular to elongated shapes. The slug flow mainly occurs in T-shaped microchannel flow boiling under high mass flow density and low heat load conditions. Under the condition of low mass flow density, the growth rate of nucleation bubbles in the branch channels is extremely fast, quickly occupying the entire bifurcation, and suppressing the vaporization core at the bifurcation. Therefore, the occurrence of the slug flow was not observed in the experimental process under low mass flow density.

#### 3.2.3. Plug–Annular Alternated Flow

The plug–annular alternating flow is a special gas–liquid two-phase flow pattern that occurs during FBM with the T-junction. Due to the bifurcations, the annular flow in the branch channel is difficult to sustain when the slug flow or plug flow exists in the main channel. The bubbles or plugs in the main channel will inevitably disrupt the annular flow liquid film in the branch channel when passing through the bifurcation, resulting in the alternate presence of plug and annular flows at the bifurcation, which is the plug–annular alternated flow pattern.

The main characteristics of the plug–annular alternated flow pattern are as follows: circular small bubbles and slug bubbles are randomly distributed in the main channel, while annular flow exists in the branch channel, and slug bubbles may also appear at the end. There are two reasons for the appearance of slug bubbles at the end of the branch channel: one reason is that the circular small bubbles in the main channel exist independently until flowing into the branch channel; another reason is that when the branch channel is in an annular flow, the vaporization core on the branch channel wall is suppressed. When the annular flow in the branch channel is disrupted, the inhibitory influence of the liquid film on the vaporization core disappears, and some nucleation bubbles will reappear in the branch channel. These nucleation bubbles quickly grow, and some of them merge with the plugs in the branch channel, while others grow independently into slug bubbles. This is the reason why slug bubbles sometimes appear at the end of the branch channel.

**Figure 19:**
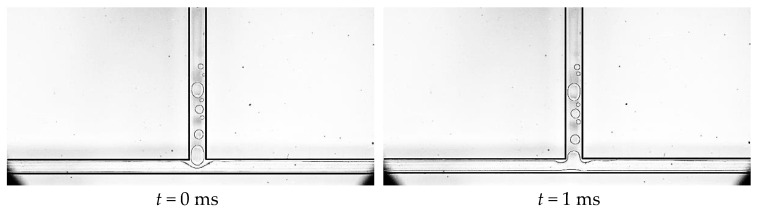

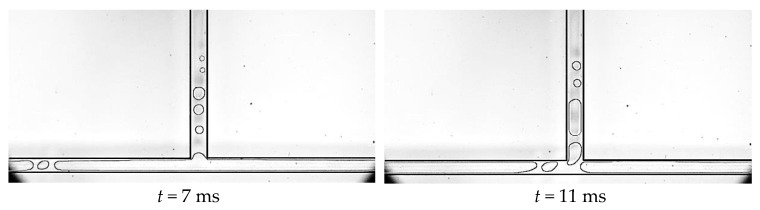
Plug–annular alternated flow, working condition: Type 2, *G* = 113.6 kg/m^2^·s and *T*_heat_ = 55.7 °C.

In the main channel, slug bubbles and circular small bubbles are irregularly distributed along the main flow direction, and the bubble size does not increase sequentially. We can observe from Figure 19 that the channel end has circular bubbles, but slug bubbles also exist in the middle, which is caused by the random merging of bubbles during the flow process. When the bubbles in the main channel pass through the bifurcation in the form of slug bubbles, there will be a merging situation with the annular flow in the branch channel. At this time, the annular flow in the branch channel is not disrupted, but a necking section appears at the bifurcation. The front end of the slug bubble merges with it before the necking section breaks, and the branch channel eventually returns to the annular flow. When the bubbles in the main channel pass through the bifurcation in the form of circular small bubbles, similar to the squeeze rupture flow pattern, the annular flow liquid film is blocked at the bifurcation. Under the compression of circular small bubbles and slug bubbles in the main channel, a necking section is formed in the liquid film until the film finally ruptures, and the annular flow gas in the branch channel is divided into two parts. The circular small bubbles and slug bubbles in the main channel quickly grow and merge after passing through the bifurcation, and most of the bubbles merge with the broken gas band in the branch channel to form a new annular flow, while a small part of the bubbles form a plug flow and flow directly to the outlet. We call this flow pattern, with an alternate presence of plug and annular flows at the bifurcation, the plug–annular alternated flow.

#### 3.2.4. Annular Flow

The annular flow is a typical two-phase flow pattern that occurs during FBM, and it is also one of the most common flow patterns in T-shaped microchannels during flow boiling. In our experiments, we observed two types of annular flow patterns: An intermittent annular flow and stable annular flow. The intermittent annular flow refers to the continuous presence of annular flow at the bifurcation point, which is always covered by a liquid film. A stable annular flow refers to the continuous presence of annular flow not only at the bifurcation point but also throughout the entire T-shaped microchannel.

**Figure 20:**
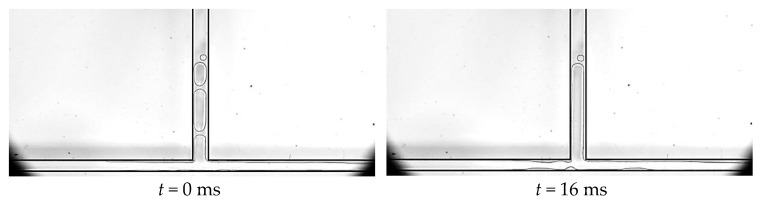
Intermittent annular flow, working condition: Type 2, *G* = 14.2 kg/m^2^·s and *T*_heat_ = 42.5 °C.

The main flow characteristics of an intermittent annular flow are as follows: slug bubbles and annular flow alternate in the main channel, while the branch channel maintains a continuous annular flow that extends to the end of the main channel, as can be observed in Figure 20. In the experiments, nucleate bubbles are generated at the upper-middle position of the main channel, and these nucleate bubbles quickly grow into slug bubbles after leaving the nucleation point. The length of slug bubbles increases sequentially in the flow direction, and during the flow process, the slug bubbles merge with each other to form longer plugs. The plugs periodically merge with the annular flow in the branch channel before reaching the bifurcation point. Throughout the experiments, no annular flow rupture phenomenon similar to the slug–annular flow pattern occurs at the bifurcation point. The merging of plugs in the main channel and the annular flow in the branch results in the annular flow throughout the entire T-shaped microchannel. However, the nucleate bubbles in the upper-middle position disrupt the annular flow in the main channel, resulting in an unstable annular flow throughout the entire T-shaped microchannel. Despite this, the bifurcation point maintains an annular flow state throughout the process, so we refer to this flow pattern as the intermittent annular flow.

**Figure 21:**
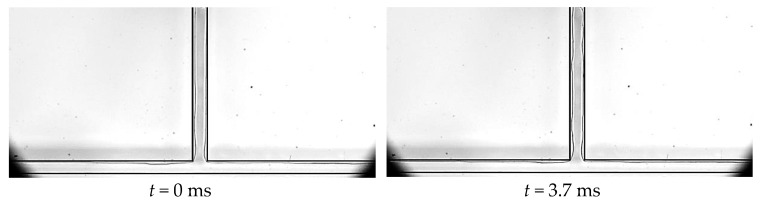
Steady annular flow, working condition: Type 2, *G* = 14.2 kg/m^2^·s and *T*_heat_ = 53.7 °C.

In contrast to the intermittent annular flow, the stable annular flow maintains a stable annular flow pattern throughout the entire T-shaped microchannel during the flow process. Its flow characteristics are as follows: the main channel and branch channel continuously maintain an annular flow pattern. Most of the liquid quickly vaporizes after entering the entrance of the main channel (as presented in Figure 6) and flows towards the outlet through the annular gas film, while a small amount of unvaporized liquid flows towards the outlet along the annular liquid film. Because of the bifurcation, the liquid flowing accumulates at the bifurcation, resulting in an increase in film thickness at the end of the main channel, as can be seen in Figure 21. Throughout the flow process, the bifurcation point maintains an annular flow state without rupture, coalescence, or other phenomena, and the entire T-shaped microchannel maintains a stable annular flow. We refer to this flow pattern as the stable annular flow. As the heating temperature continues to rise, the annular liquid film evaporates continuously, causing it to become thinner and eventually disappear completely, leading to a drying-out phenomenon.

### 3.3. Flow Boiling Flow Pattern at the Bifurcation of T-Shaped Microchannel and Its Influencing Factors

**Figure 22:**
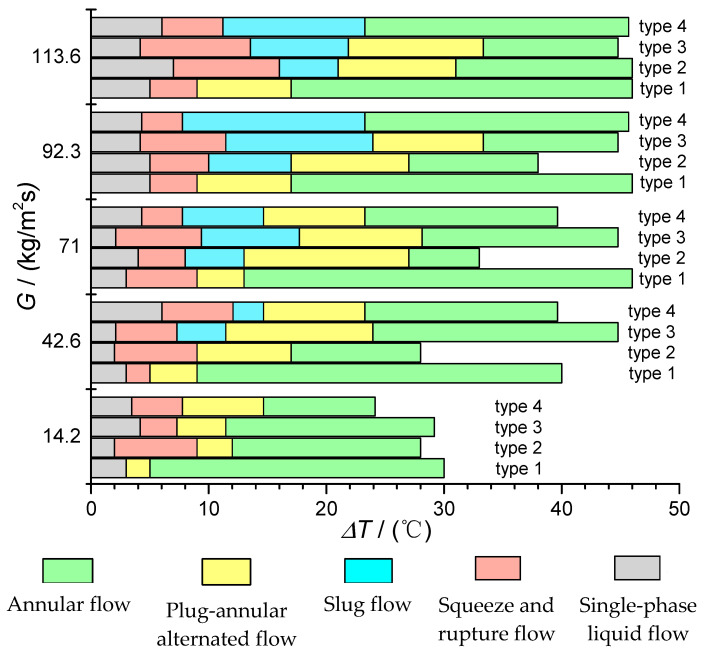
Flow boiling flow pattern at the bifurcation of a T-shaped microchannel.

The experiment was conducted on T-shaped microchannels of four different sizes to study flow boiling. During the experiment, the operating conditions were changed by adjusting the mass flow rate at the channel inlet and the heating temperature of the chip. Figure 22 shows the flow regime map at the bifurcation point under different operating conditions. The *y*-axis represents the mass flow rate at the entrance of microchannel (*G*), and the *x*-axis represents the degree of the superheat of the chip (Δ*T*). As shown, the factors that primarily affect the flow mechanism at the bifurcation point of the microchannel are the mass flow rate at the inlet, the degree of superheat (Δ*T*), and the size of it. The specific effects of these three factors on the flow regime at the bifurcation point of the T-shaped microchannel will be discussed below.

#### 3.3.1. Effect of Fluid Mass Flow Density on the Flow Pattern of Flow Boiling at the Bifurcation of T-Shaped Microchannel

**Figure 23:**
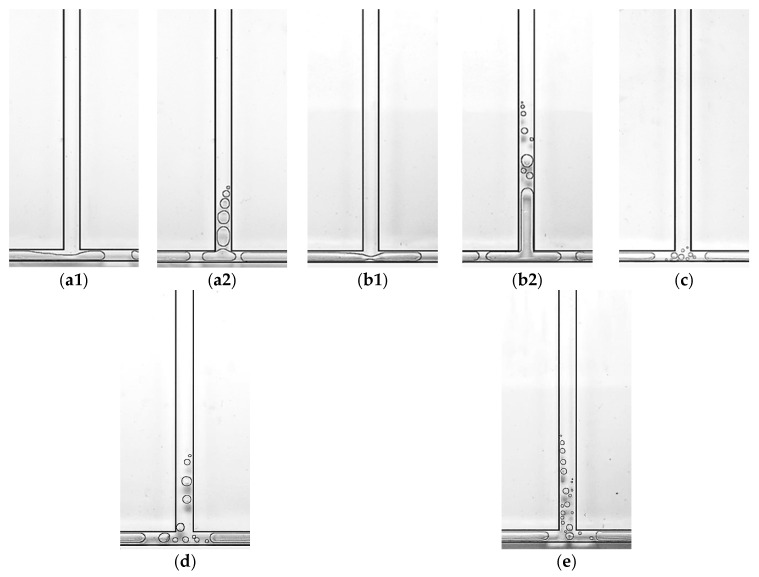
Effect of microchannel size on slug flow in T-shaped microchannel, working conditions: (**a1**) Type 1, *G* = 42.6 kg/m^2^·s and *T*_heat_ = 35.8 °C; (**a2**) type 1, *G* = 42.6 kg/m^2^·s and *T*_heat_ = 37.9 °C; (**b1**) type 2, *G* = 42.6 kg/m^2^·s and *T*_heat_ = 39.8 °C; (**b2**) type 2, *G* = 42.6 kg/m^2^·s and *T*_heat_ =41.9 °C; (**c**) type 3, *G* = 42.6 kg/m^2^·s and *T*_heat_ = 39.9 °C; (**d**) type 1, *G* = 42.6 kg/m^2^·s and *T*_heat_ = 46.7 °C; (**e**) type2, *G* = 71 kg/m^2^·s and *T*_heat_ = 41.8 °C.

This experiment studied the flow boiling behavior of chips of the same size with five different mass flux densities. As shown in Figure 23(b1,b2,e), in type 2 chips, no bubble flow was observed at the bifurcation under the condition that the mass flux density was less than 42.6 kg/m^2^·s, while the bubble flow appeared when the mass flux density was bigger than 71 kg/m^2^·s. This is because, at the same heating temperature, when the mass flux density is low, the nucleate bubbles generated in the main channel rapidly grow into slug bubbles after leaving the vaporization nucleus, and even merge and grow into slug plugs during the downstream flow; thus, the two-phase always exists in the form of slug plugs or gas bands at the bifurcation. In addition, at the minimum mass flux density of 14.2 kg/m^2^·s, the bubble flow was absent in all four chip sizes, while when the mass flux density increases, the bubble flow appeared successively in type 3, type 4 and type 2 chips, and only the type 1 chip did not show a bubble flow in this range of mass flux. This reflects the influence of the microchannel size effect.

Furthermore, in the four different chip sizes, when the mass flux density increases, the appearance time of the annular flow gradually delays, as can be observed in Figure 22. Under the condition that the mass flux density is low, the time for the slug–annular alternating flow pattern is short, and it quickly evolves into an annular flow pattern; thus, the appearance of the annular flow pattern is relatively early. The increase in mass flux density provides more subcooled liquid; thus, the previously missing bubble flow appears during the flow process, and the appearance time of the annular flow is delayed. However, at high mass flux densities, the time difference for the appearance of the annular flow gradually decreases, indicating that the influence of mass flux density on the appearance time of the annular flow gradually weakens.

#### 3.3.2. Influence of Microchannel Size on the Flow Pattern of FBM with T-Junction

The flow boiling phenomena were investigated in four different-sized chips through visualization experiments. The main channel width of the type 1, type 2, and type 3 chips is 800 μm, and the width of the branch channels is 600 μm. The depths of the three chips are 300 μm, 450 μm and 600 μm. The main channel width of the type 4 chip is 1000 μm, and the width of the branch channels is 700 μm. All of them have a depth of 800 μm. Based on the division of microchannel sizes, type 1, type 2, and type 3 chips can be regarded as microchannels, while the type 4 chip can be considered as a conventional small channel. As shown in Figure 23, under the operating condition of a mass flow rate density of 42.6 kg/m^2^·s, the flow pattern at the bifurcation of the type 1 chip is the squeeze-induced rupture flow (Figure 23(a1)), with liquid flow in the main channel and slug flow in the branch channel. With a slight increase in the heating temperature to 37.9 °C, vapor bubbles produced by nucleation appeared in the main channel, but they had grown into slugs by the time they reached the bifurcation, and no bubble flow was observed at the bifurcation (Figure 23(a2)). Similarly, at a heating temperature of 37.9 °C, the bifurcation of the type 2 chip exhibited squeeze-induced rupture flow (Figure 23(b1)). As the heating temperature increased to 39.8 °C, the bifurcation displayed a slug–annular alternating flow (Figure 23(b2)). The main reason for this phenomenon is that, under a mass flow rate density of 42.6 kg/m^2^·s, the nucleation bubbles generated in the main channel of type 1 and type 2 chips quickly grow into slugs because of the channel size limitation, and the two-phase flow at the bifurcation directly transforms from a single-phase liquid to slugs. Therefore, the bubble flow pattern is absent at the bifurcation. On the contrary, due to the greater depth of the type 3 chip, the growth of nucleation bubbles in the main channel is limited in the depth direction compared to type 1 and type 2 chips. Hence, small bubbles can be clearly observed at the bifurcation at a heating temperature of 39.9 °C (Figure 23c). Similarly, the bubble flow at the bifurcation is also more pronounced in the type 4 chip (Figure 23d). At mass flow rate densities of 71, 92.3 and 113.6 kg/m^2^·s, bubble flow was only absent at the bifurcation of the type 1 chip, while it was observed at the bifurcation of the other three chips. The absence of bubble flow under relatively high mass flow rate densities further demonstrates the effect of microchannel size on the flow pattern.

The limitation of microchannel size contributes to the absence of bubble flow in shallow-depth chips, because the two-phase flow in the restricted channel exists as small bubbles for a very short time or even directly transforms from a single-phase liquid to slugs. At the same time, due to the limitation of the channel size, the appearance time of the annular flow will also change. As shown in the flow pattern diagram in Figure 22, under a constant mass flow rate density, such as 92.3 kg/m^2^·s, the annular flow first appears in the type 1 chip and last appears in the type 3 chip. Therefore, reducing the size of microchannels will cause the annular flow to appear earlier.

### 3.4. Unstable Outlet Temperature in T-Shaped Microchannels

Flow instability is a key issue in microscale flow and heat transfer research, and temperature instability is the most common research content. During the experiments, the inlet temperature of the supercooled liquid in the main channel was kept constant, and the instability of the outlet temperature was studied by changing the heating temperature and liquid flow rate. Experimental results show that both the heating temperature as well as the mass flow rate density of the inlet liquid have an influence on the fluctuation of the outlet temperature.

#### 3.4.1. Influence of Heating Temperature on Outlet Temperature Instability

As shown in Figure 22, under different heating temperatures, the bifurcation of the T-shaped microchannel exhibits different flow patterns. Therefore, the effect of the heating temperature on the instability of the outlet temperature reflects, to some extent, the influence of different flow patterns on the instability of the outlet temperature. For a qualitative and quantitative analysis, we selected the type 1 chip and analyzed the operating condition with a main channel inlet flow rate of 113.6 kg/m^2^·s. When the heating temperatures were 35.8 °C, 41.9 °C, 50 °C and 62 °C, as shown in Figure 22, the corresponding flow patterns at the bifurcation of the T-shaped microchannel were the squeeze-induced rupture flow, slug–annular alternating flow, unstable annular flow and stable annular flow. 

**Figure 24:**
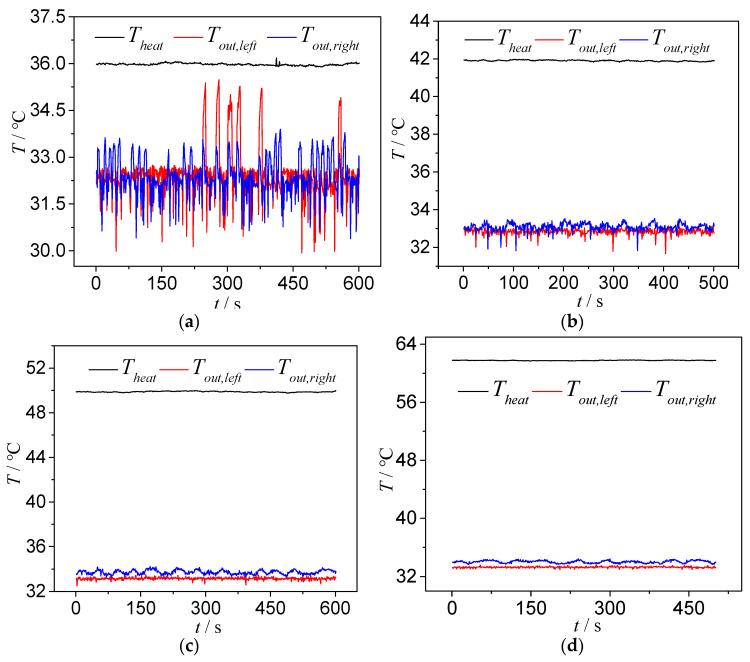
Outlet temperature fluctuations at different heating temperatures: type 1, *G* = 113.6 kg/m^2^·s. (**a**) Type 1, *G* = 113.6 kg/m^2^·s and *T*_heat_ = 35.8 °C; (**b**) type 1, *G* = 113.6 kg/m^2^·s, *T*_heat_ = 41.9 °C; (**c**) type 1, *G* = 113.6 kg/m^2^·s and *T*_heat_ = 50 °C; (**d**) type1, *G* = 113.6 kg/m^2^·s and *T*_heat_ = 62 °C.

**Figure 25:**
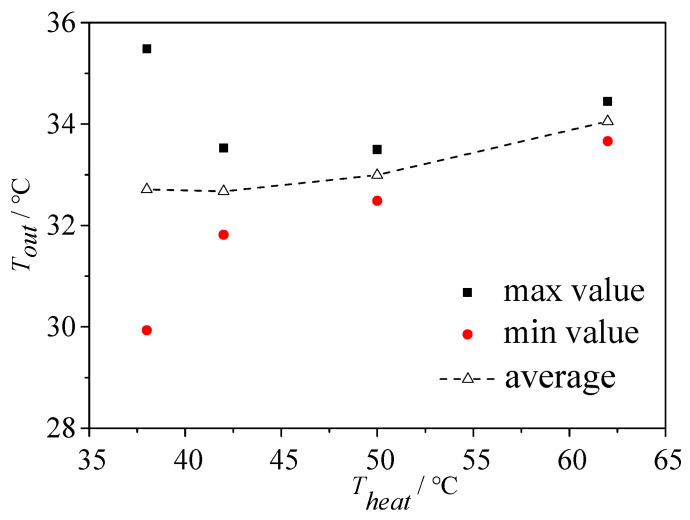
Outlet temperature at different heating temperatures: Type 1, *G* = 113.6 kg/m^2^·s.

When the heating temperature is 35.8 °C, the bifurcation of the T-shaped microchannel exhibits a squeeze-induced rupture flow, with a liquid flow in the main channel and a pulsating slug flow in the branch channel. The slug flow will be squeezed and ruptured into two sub-slugs at the bifurcation. Therefore, at the left and right outlets of the microchannel, sometimes there is a single-phase supercooled liquid flow and sometimes there is a slug flow, with a small void fraction. This leads to the fluctuation of the temperatures at the left and right outlets, as shown in Figure 24a. The fluctuation of the outlet temperature is more pronounced due to the fluctuation of the outlet pressure (caused by the rupture of slugs at the outlet, etc.), which leads to the pulsating slug flow in the branch channel having a significantly increased residence time, allowing more sufficient heat exchange between the gas phase in the slug wall. The pulsating slug flow in the branch channel is the main reason for the more intense fluctuation of the outlet temperature in the squeeze-induced rupture flow. Figure 25 shows the fluctuation of the outlet temperature of the type 1 chip under a different heat load. We can conclude that the variations of the outlet temperature reduce when the heating temperature increases.

As the heating temperature increases, as presented in Figure 24b and with the decrease in heating temperature by 41.9 °C, the bifurcation of the T-shaped microchannel exhibits a plug-ring alternating flow. The inlet section is still a subcooled single-phase liquid flow, while the end of the main channel is mainly a slug or plug flow, with the plug and annular flow alternating in the branch channel. The bubbles (plugs) in the main channel split into two sub-bubbles (plugs) at the bifurcation, which drives the two-phase flow in the left and right branch channels, eliminating the pulsation phenomenon of the plug flow and squeezing in the broken flow. All the bubbles (plugs) flow directly to the outlet, reducing the relative heat exchange time between the gas and the microchannel. Therefore, the peak change of the outlet temperature decreases, and at the same time, due to the increase in the proportion of the outlet void, the fluctuation amplitude of the outlet temperature decreases.

When the heating temperature further increases, as presented in Figure 24c,d, when the heating temperature reaches 50 °C and 62 °C, the bifurcation of the T-shaped microchannel shows the annular flow. The bifurcation and the branch channel maintain the annular flow, and the void fraction at the outlet approaches one. At this time, the variations of the outlet temperature are further weakened. The difference is that at a heating temperature of 50 °C, it is unstable annular flow, and the annular flow at the outlet fluctuates. At a heating temperature of 62 °C, it is stable as the outlet becomes thinner and relatively stable; thus, the fluctuation of the outlet temperature is relatively reduced.

#### 3.4.2. Effect of Mass Flow Density on Outlet Temperature Instability

**Figure 26:**
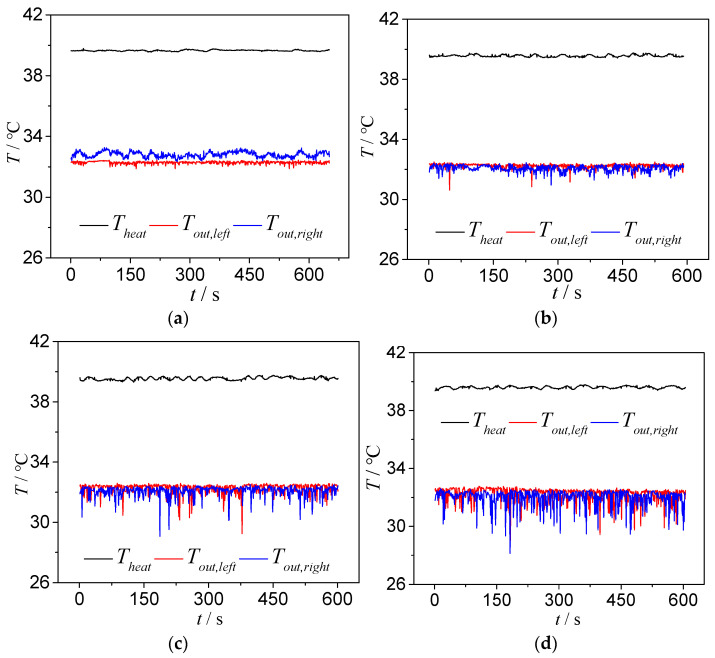
Outlet temperature fluctuations under different mass flow density: type 3, *T*_heat_ = 39.6 °C. (**a**) Type 3, *G* = 42.6 kg/m^2^·s and *T*_heat_ = 39.6 °C; (**b**) type 3, *G* = 71 kg/m^2^·s and *T*_heat_ = 39.6 °C; (**c**) Type 3, *G* = 92.3 kg/m^2^·s and *T*_heat_ = 39.5 °C; (**d**) type 3, *G* = 113.6 kg/m^2^·s and *T*_heat_ = 39.6 °C.

**Figure 27:**
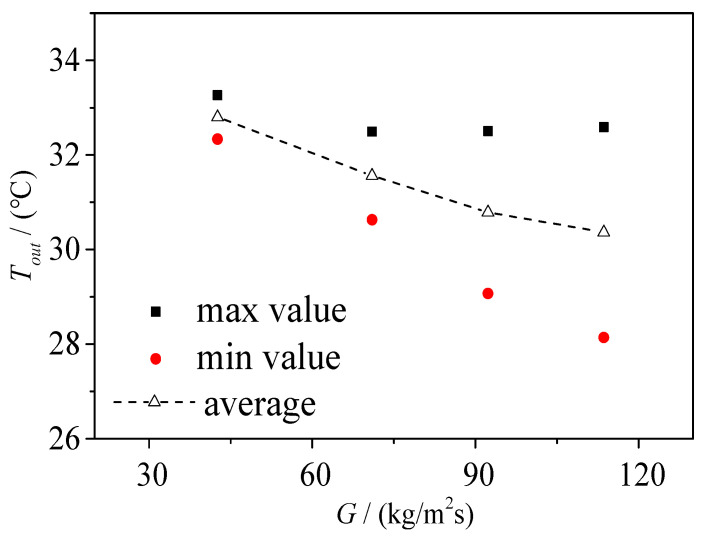
Outlet temperature under different mass flow density: type 3, *T*_heat_ = 39.6 °C.

From Section 3.3.1, we can find that that the mass flow rate density of the inlet exerts a vital impact on the flow regime of FBM with the T-junction, which is consistent with the flow boiling regime diagram at the bifurcation in Figure 22. As shown in Figure 26, in the type 3 chip, when the heating temperature is 39.6 °C, the temperature at the left and right outlets fluctuates when the inlet mass flow rate densities are around 42.6 kg/m^2^·s, 71 kg/m^2^·s, 92.3 kg/m^2^·s, and 113.6 kg/m^2^·s. Figure 27 presents the temperature fluctuation range at the left and right outlets under different mass flow rate densities. The results show that around the heating temperature of 39.6 °C, with the increase in the mass flow rate density, the temperature fluctuation range at the left and right branches gradually increases, but the average temperature at the outlets decreases.

As shown in Figure 22, in the type 3 chip, when the heating temperature is 39.6 °C, the flow regimes at the bifurcation correspond to a slug–annular alternating flow, bubble flow, squeeze and rupture flow, and squeeze and rupture flow for inlet mass flow rate densities of 42.6 kg/m^2^·s, 71 kg/m^2^·s, 92.3 kg/m^2^·s, and 113.6 kg/m^2^·s, respectively. Similar to the impact of heating temperature on outlet temperature instability, the influence of the mass flow rate density on the outlet temperature instability is ultimately reflected in the influence of flow regimes on the outlet temperature instability. In the type 3 chip, when the heating temperature is 39.6 °C and the inlet mass flow rate density is 42.6 kg/m^2^·s, the flow regime at the bifurcation is the slug–annular alternating flow. The left and right outlets are occupied alternately by slugs and gas regions. At this moment, the void fraction at the outlet cross-section is relatively large and mainly occupied by the gas phase. Therefore, the temperature fluctuation range at the left and right outlets is relatively small, as can be observed in Figure 26a.

We can find in Figure 26b and Figure 26 that, when the mass flow rate density increases to 71 kg/m^2^·s, at a heating temperature of 39.6 °C, the flow regime at the bifurcation in the type 3 chip becomes the bubble flow. At this time, the flow through the left and right outlets is mainly composed of bubbles, slugs, and subcooled liquid. Since a small amount of subcooled liquid passes through the left and right outlets, the minimum value of the temperature fluctuation at the outlets is significantly lower than that at a mass flow rate density of 42.6 kg/m^2^·s and lower than the saturation temperature of 32.05 °C.

When the mass flow rate density further increases to above 92.3 kg/m^2^·s, as presented in Figure 26c,d, the temperature fluctuation range at the inlet and outlet further increases. This is because the flow regime at the bifurcation in the type 3 chip becomes a squeeze and rupture flow. At this time, the void fraction at the cross-section is smaller, and the flow through the left and right outlets is mainly composed of slugs and subcooled liquid. Moreover, for the same period of time, the proportion of the subcooled liquid flow is larger compared to that in the bubble flow regime. This is determined by the characteristics of the squeeze and rupture flow regime, where the slug oscillates back and forth in the branch channel, and most of the flow through the outlet is subcooled liquid after the oscillating slug is squeezed out at the bifurcation. This alternating flow of slugs and subcooled liquid through the outlet is the main reason for the largest amplitude of temperature fluctuation at the left and right outlets.

## 4. Conclusions

To study the flow boiling phenomenon and mechanism in bifurcated microchannels, this chapter designs and fabricates four different-sized T-shaped microchannel chips, and establishes an experimental platform for flow boiling in T-shaped microchannels. The flow patterns of FLM with the T-junction can be observed through visualization experiments, and the variation of flow patterns and influencing factors in four different-sized T-shaped microchannels under different operating conditions are analyzed. Moreover, the temperature instability of FBM with the T-junction is also investigated, and the experimental results show that:

(1) In the flow boiling of T-shaped microchannels, the vaporization nuclei first appear in the branch channels, and as the heating temperature increases, the vaporization nuclei gradually extend into the main channel.
(2) The flow patterns of FBM with the T-junction mainly include the squeeze and rupture flow, slug flow, plug–annular alternating flow, and annular flow.
(3) Apart from the heating temperature, the inlet mass flow rate also affects flow patterns at the bifurcation of microchannels. When the mass flow rate at the entrance decreases, the bubble flow at the bifurcation disappears, and when the inlet mass flow rate increases, the appearance of the annular flow at the bifurcation is delayed. Furthermore, the size of the T-shaped microchannel also has an influence on the flow boiling flow patterns at the bifurcation. The bubble flow disappears in smaller microchannels, and with the increase in microchannel size, the timing of the appearance of the annular flow at the bifurcation is also delayed.
(4) Flow boiling in T-shaped microchannels results in temperature instability at the outlet, which mainly manifested as temperature fluctuations. The heating temperature and outlet mass flow rate affect the variations of the outlet temperature. As the heating temperature increases, the variations of the outlet temperature gradually weaken. Conversely, as the mass flow rate at the entrance increases, the variations of the outlet temperature gradually weaken. These two influencing factors ultimately stem from the effect of flow patterns on temperature fluctuations at the outlet.
(5) Future studies can be carried out from the following aspects: (1) Flow boiling phenomenon of different working fluids in bifurcated microchannels, and a more applicable flow pattern diagram, are established. (2) Thickness change of the bubble necking section in the nitrogen–water two-phase flow at a high mass flow density. (3) Effects of bifurcation angle and shape on gas–liquid two-phase flow in a bifurcated microchannel. The research work in this paper will help to understand the flow boiling two-phase flow characteristics in complex microchannel networks. With the increase in electronic device layout density, the T-type microchannel structure will be a method to solve the high heat flux of electronic devices.

## Figures and Tables

**Table 1:** Dimensions of four T-shaped microchannels.

	Type 1	Type 2	Type 3	Type 4
*L*_1_ (mm)	32	32	32	32
*L*_2_ (mm)	24	24	24	24
*w*_1_ (μm)	800 (857)	800 (877)	800 (898)	1000 (1122)
*w*_2_ (μm)	600 (796)	600 (694)	600 (673)	700 (837)
*h* (μm)	300 (367)	450 (551)	600 (673)	800 (939)
*D*_1_	436 (514)	576 (676)	686 (769)	888.9 (1022)
Note: The value in brackets is the actual value measured under the microscope, such as 800 (857); the design size is 800 μm; the actual size is 857 μm.				
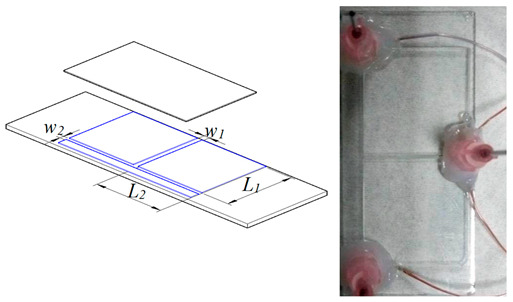				

**Table 2:** Information about the instruments and equipment used in the experiment.

No.	Instrument	Type, Equipment Manufacturers and Main Parameters
1	Injection pump	Type: LSP01-1BH, Baoding Lange Constant Flow Pump Co., Ltd. (Baoding, China); comes with 50 mL stainless steel syringe
3	High-speed camera	Type: Photron FASTCAM SA4, Photron Co., Ltd. in Japan (Tokyo, Japan)Maximum frame rate: 500,000 fps; Resolution ratio: 1024 × 1024 (3600 fps)
4	Microscope	Type: AF Micro-Nikkor 60 mm F/2.8D NIKON (Tokyo, Japan)
5	High frequency no flashing light	Type: DAK-C1-0408, Foshan Dongxiong lighting factory (Foshan, China)
7	Three-dimensional mobile platform	Type: LSSP-125WS Translation table, Jiangxi Liansheng Technology Co., Ltd. (Nanchang, China)
8	DC power supply	Type: KORAD KA3005D, Shenzhen Kerui source Technology Co., Ltd. (Shenzhen, China)
9	Optical experimental platform	Type: POT24-12, Jiangxi Liansheng Technology Co., Ltd.
10	Agilent data acquisition device	Type: Agilent 34970a, Agilent Technology Co., Ltd. (Santa Clara, CA, USA), equipped with copperConstantan thermocouple produced by American OMEGA Company (Norcross, GA, USA), that is, T-shaped thermocouple
11	Thermostatic waterbath	Type: JULABO MC, Beijing Youlebo Technology Co., Ltd. (Beijing, China)

## Data Availability

Data are contained within the article.
